# HIV but Not CMV Replication Alters the Blood Cytokine Network during Early HIV Infection in Men

**DOI:** 10.3390/v14081833

**Published:** 2022-08-21

**Authors:** Christophe Vanpouille, Alan Wells, Jennifer M. Dan, Stephen A. Rawlings, Susan Little, Wendy Fitzgerald, Leonid Margolis, Sara Gianella

**Affiliations:** 1 Eunice Kennedy Shriver National Institute of Child Health and Human Development, National Institutes of Health, Bethesda, MD 20892, USA; 2 Department of Medicine, University of California San Diego, La Jolla, CA 92161, USA

**Keywords:** HIV, CMV, cytokine, semen, blood, MSM, PLS-DA

## Abstract

Objective: CMV coinfection contributes to sustained immune activation in people with chronic HIV. In particular, asymptomatic CMV shedding in semen has been associated with increased local and systemic immune activation, even during suppressive antiretroviral therapy (ART). However, the effect of seminal CMV shedding in people with HIV in the earliest phase of HIV infection is not known. Methods: Using Luminex, we measured the concentration of 34 cytokines in the blood plasma of sixty-nine men who had sex with men with or without HIV and in subgroups of CMV shedders vs. non-shedders. Differences in blood plasma cytokines between groups were investigated using the multivariate supervised partial least squares discriminant analysis method. Results: Independently of CMV, we found that concentrations of IP-10, MIG, MCP-1, I-TAC 10, IL-16, and MIP-1β were modulated in the earliest phase of HIV infection compared with control individuals without HIV. In people with HIV, there was no difference in blood cytokines among CMV shedders vs. non-shedders. Conclusion: In early/acute HIV infection, asymptomatic CMV shedding in semen does not drive additional cytokine changes in blood. Early ART initiation should remain the priority, while the added benefit of CMV suppression during the various stages of HIV infection needs to be further investigated.

## 1. Introduction

Although antiretroviral therapy (ART) significantly improves the lifespan and general health of people with HIV (PWH), they remain at risk of developing non-AIDS comorbidities, such as end-stage organ disease and age-related diseases (reviewed in [1,2]). Increased morbidity and mortality in PWH are associated with inflammation and immune dysfunction, which persist even despite suppressive ART [3,4]. The reasons for persistent immune activation, a driving force of HIV disease, are likely multifactorial (reviewed in [5]). Besides low-level HIV replication, loss of regulatory cells, and gut damage resulting in bacterial and fungal translocation, CMV coinfection has been proposed as a key player in sustaining immune activation (reviewed in [6]).

CMV is a ubiquitous β-herpesvirus establishing lifelong infection through latency with periodic subclinical reactivation [7]. Although benign in heathy individuals, CMV exacerbates the development of HIV-triggered immunological abnormalities (reviewed in [5,8,9]) in PWH, amongst whom CMV prevalence is about 90% [10].

Despite ART, CMV seropositivity is associated with higher levels of differentiated CD4^+^ and CD8^+^ T-cells, leading to accelerated T-cell immunosenescence and ultimately immune exhaustion [11,12,13,14,15]. Strong associations have also been found between CMV IgG antibody levels and soluble markers of immune activation, such as C-reactive protein (CRP), sCD163 tumor necrosis factor–alpha, IL-6, and sCD14 [16,17,18,19,20,21].

PWH are more likely than the general population to have subclinical bursts of CMV replication at mucosal sites and in semen. CMV shedding in semen has been associated with increased local [22] and systemic [11,23,24] immune activation in people with chronic HIV and those suppressed on ART. However, the effect of asymptomatic CMV shedding in semen on immune activation in PWH in early/acute HIV infection has not been reported. If CMV replication were found to alter the cytokine network during early HIV infection, treatment of CMV in addition to ART could prevent subsequent immune disfunction, similar to valacyclovir for herpes simplex virus in chronic HIV [11]. Here, we investigated the effect of seminal CMV shedding (as a proxy of asymptomatic CMV replication) on systemic cytokine production in a cohort of PWH with early/acute infection.

Using the multivariate supervised partial least squares discriminant analysis (PLS-DA) statistical method, we found that early HIV infection is associated with cytokine changes in blood plasma, while the presence of CMV genital shedding is not associated with any systemic changes in cytokines.

## 2. Methods

### 2.1. Study Participants

Blood plasma samples from 69 individuals (48 PWH from the San Diego Primary Infection Cohort and 21 without HIV but with similar HIV risk factors from a control cohort) were analyzed for cytokine expression [22,25]. Blood plasma cytokines were analyzed for all 69 individuals. The study was conducted according to the guidelines of the Declaration of Helsinki and approved by the Institutional Review Board of the Human Research Protections Program at the University of California, San Diego (protocol code no.: 191088; approval date release: 8 June 2021). Informed consent was obtained from all subjects involved in the study.

Semen was collected as previously described [26]. Among PWH, 25 had genital CMV shedding and 22 did not. CMV shedding was available only for 47 out 48 PWH. CMV DNA was quantified in semen, following previously published methods [26]. Baseline count values were cell counts at study entry.

Since clinical CD4 and CD8 data were not available for people without HIV, CD4 and CD8 percentages were calculated from a flow cytometry panel on samples taken the same day as the samples analyzed in the cytokine assay.

### 2.2. Multiplex Bead Array Assay for Cytokine/Chemokine Quantification

(i) The National Institutes of Health laboratory, part of the Microbicide Quality Assurance Program, performed Luminex measurements for 34 cytokines/chemokines involved in different immunological functions (Table S1) [27].
(ii) Mediators of innate immunity, inflammation, and chemotaxis (Interleukin (IL)-1α, IL-1β, IL-6, IL-17, IL-18, IL-21, IL-22, IL-33, Cal, IL-8/CXCL8, MIG/CXCL9, IFN-inducible protein (IP)-10/CXCL10, I-TAC/CXCL11, TNF-α, monocyte chemotactic protein (MCP)-1/CCL2, macrophage inflammatory protein (MIP)-1α/CCL3, MIP-1β/CCL4, regulated on activation, normally T-cell expressed and secreted (RANTES/CCL5), Eotaxin/CCL11, MIP-3α/CCL20, and GRO-α/CXCL1.
(iii) Mediators of hematopoiesis: macrophage colony-stimulating factor (M-CSF) and granulocyte macrophage colony-stimulating factor (GM-CSF)).
(iv) Anti-inflammatory cytokines: IL-10, IL-13, and transforming growth factor (TGF)-β.
(v) Mediators of lymphocytes activation, proliferation, and differentiation: IL-2, IL-4, IL-7, IL-12, IL-15, IL-16, CCL3, CCL4, CCL5, CCL20, and IFN-γ.
(vi) Human CMV IL-10 homolog (cmvIl-10).

Bead coupling was prepared according to the manufacturer’s recommendations. All standards and capture and detection antibodies were purchased from R&D (Minneapolis, MN, USA), except for IL-4 (Biolegend, San Diego, CA, USA), IL-12 (BD Biosciences, Franklin Lakes, NJ, USA), and IL-21 (Thermo Fisher, Waltham, MA, USA).

### 2.3. Statistical Analysis

Cytokines that were undetectable >70% of the time or more were excluded from the analysis. Undetectable cytokine values were replaced by the minimum of half the lower limit of detection. The limit of detection for each cytokine is provided in the Supplementary Materials. Log-transformed concentrations of cytokines were used for PLS-DA, as previously described [28]. PLS-DA models are particularly suitable when predictors (e.g., cytokines) have more variables than observations (here, HIV serostatus and CMV shedding status). PLS-DA allowed us to visualize the separation in the cytokine profiles between PWH and controls without HIV infection. In the subpopulation of PWH, PLS-DA allowed us to visualize the separation between individuals shedding CMV in their semen and those who did not. In addition, we fit a PLS-DA model, including all 3 groups (people without HIV, CMV shedders, and non-shedders among people with early/acute HIV infection).

The classification performance of the PLS-DA model was assessed with the perf function using 5-fold cross-validation repeated 100 times. From the performance results, a 2-component model was used for the PLS-DA model, as the performance and number of components necessary for the final model dropped off with 3 components or more. The difference in the PLS projections was measured using the E-statistic for a 2-sample difference in the multivariate normal distribution [29]. When PLS projections were different, the difference for each cytokine with variable importance in projection (VIP) >1 was further tested with the Wilcoxon signed-rank test. To correct for multiple comparisons, raw *p*-values were adjusted using the Benjamini–Hochberg procedure. Analyses were performed using R 4.1 (R core Team, Vienna, Austria) [30], the E-test was performed using the “energy” package [31], and the PLS-DA was performed using the “mixOmics” package [32].

In addition to the multivariate statistical analyses described above, cytokines were compared individually using a Wilcoxon rank-sum test to test for differences in median concentrations between groups analyzed by PLS-DA. In this confirmatory analysis, where each cytokine was analyzed individually, *p*-values, adjusted using the FDR method, were different to the adjusted *p*-values obtained in the PLS-DA method due to the larger number of comparisons.

## 3. Results

### 3.1. Participants, Samples, and Clinical Laboratory Findings

The cohort characteristics are summarized in Table 1. All 69 individuals included in this study were men originally enrolled in a cohort of individuals at high risk for HIV infection. Out of these 69 individuals, 21 remained seronegative and 48 seroconverted to HIV with a median estimated time of infection of about 12 weeks. There was no age difference between people with and without HIV, but there were significant differences in the mean values for CD4 and CD8 percentages. All PWH were men who had sex with men (MSM) off antiretroviral therapy. Among people without HIV, 81.0% were MSM and 28.6% received pre-exposure prophylaxis. PWH were mostly White (59.6%), Hispanic/Latino (21.3%), or Other/Multiracial (19.1%). No race or ethnicity data was available for people without HIV.

Among PWH, 53.2% (n = 23) had detectable CMV in their semen, while 22 (46.8%) did not. The characteristics of people shedding CMV in semen or not are summarized in Table 2. Race, ethnicity, age, gender, CD4 T-cell counts, CD8 percentage at cytokine sample date, and HIV RNA levels were not statistically different between CMV shedders and non-shedders. CD4 percentage at cytokine sample date was significantly different between CMV shedders and non-shedders. The median CMV viral load among CMV shedders was 4.73 log_10_ copies/mL.

Thirty-four cytokines/chemokines (see Section 2) were measured in the blood of all 69 individuals. Differences in the concentrations of these markers were reported (i) for PWH in comparison to people living without HIV and (ii) for CMV shedders versus non-shedders among PWH.

### 3.2. Cytokine Profile in the Blood of People with Early/Acute HIV Is Different from That of Controls without HIV

**Figure 1:**
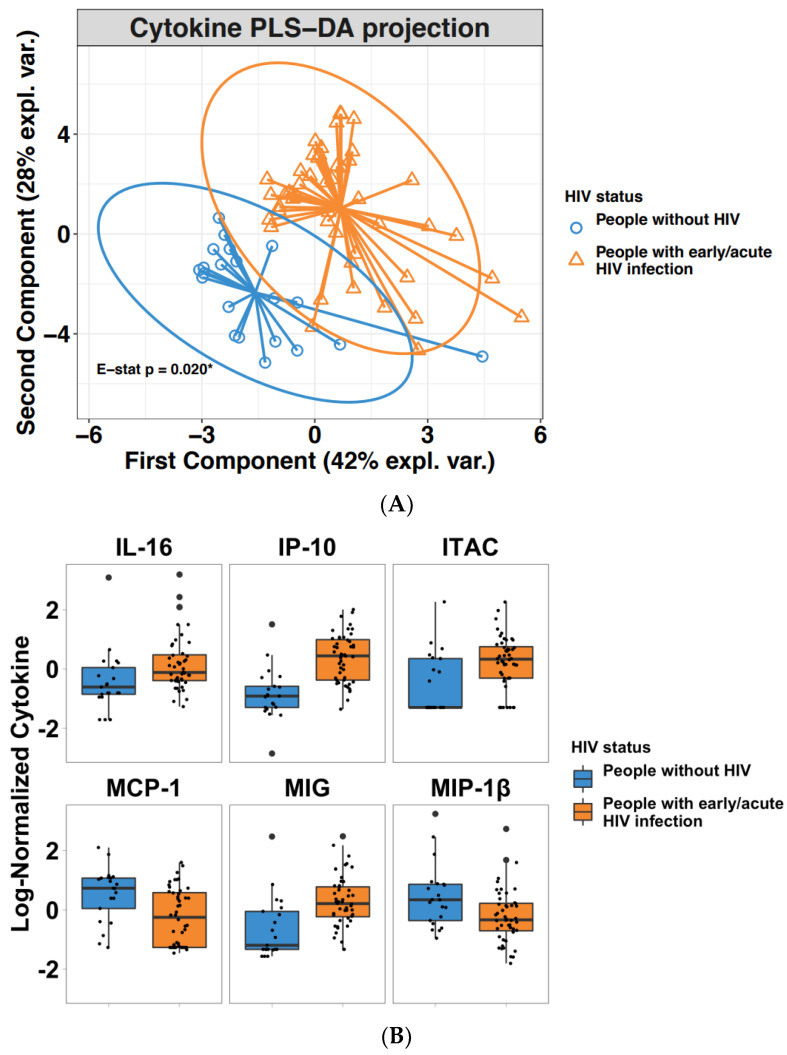
(**A**) Two-dimensional PLS projections of blood cytokines in people with and without HIV. Shown are PLS-DA projections in two LVs with ellipses representing Hotelling’s 2-samples T2 with 95% confidence intervals in blood plasma for people with early/acute HIV (orange triangles) or without HIV (blue circles). The E-statistic was used to test the statistical differences in the separation between the cytokine profiles of the two groups. The multivariate distance between people with and without HIV was significant (*p* = 0.02). (**B**) Effect of early/acute HIV infection on chemokines/cytokines in blood. The statistical significance of 12 cytokines with VIP > 1 identified in the PLS-DA model were tested by Wilcoxon Rank Sum test. Six out of 12 remained significant. Shown is the difference of the log10-transformed concentrations of the 6 chemokine/cytokines in people with or without HIV and plotted as boxplots. For each cytokine and each boxplot, each point represents a participant’s cytokine concentration, the box represents the interquartile range (IQR), the middle line represents the median, while the points beyond the whiskers are outliers.

We used the multivariate supervised PLS-DA statistical method to investigate the differences in blood cytokines between people with and without HIV. PLS-DA, which has demonstrated great success in modelling high-dimensional datasets to predict outcome, works by reducing the number of variables. In our study, the optimal model was obtained with two components or latent variables (LVs), with an error rate of 12% (Figure S1). As a result, our model suggests that blood cytokine values can predict early/acute HIV infection in PWH in comparison to people without HIV with 88% accuracy. Samples projected into the subspace spanned by the two LVs are shown as PLS projections (Figure 1A). LV1 explains 42% of variance in cytokines and 100% in HIV status; LV2 explains 28% of variance in cytokines and 69% in HIV status.

PLS projections showed distinct separation between the cytokine profiles in people with HIV versus people without HIV. Confidence ellipses for each class were plotted to highlight the strength of the discrimination (confidence level set to 95%). The separation between the two groups, measured as the energy (E) statistic, was statistically different (E-statistic *p* = 0.02) (Figure 1A).

In our PLS-DA model, each cytokine was assigned a weight or loading for both LV1 and LV2. These loadings were used to define a VIP score, which reflects the importance of a given cytokine. Cytokines with VIP > 1, which were deemed important to predict acute HIV infection, were IP-10, MCP-1, MIP-1β GM-CSF, MIG, IL-18, I-TAC, IL-17, RANTES, IL-16, eotaxin, and TNFα (in descending order) (Table 3 and Table S2). When subjected to Wilcoxon rank-sum testing, only 6 cytokines remained significant (Table 4). These cytokines were IP-10, MIG, MCP-1, I-TAC 10, IL-16, and MIP-1β (from most to least significant). IP-10, MIG, I-TAC, and IL-16 were upregulated 2.7, 1.34, 1.17, and 1.23 times, respectively, while MCP-1 and MIP-1β were downregulated 2.35 and 1.49 times (Figure 1B). The same cytokines were found to be statistically significant between people with early/acute HIV and people without HIV in an independent individual cytokine analysis (Table S3).

### 3.3. Seminal CMV Shedding Does Not Impact Blood Plasma Cytokines of People Living with HIV during Early Infection

**Figure 2:**
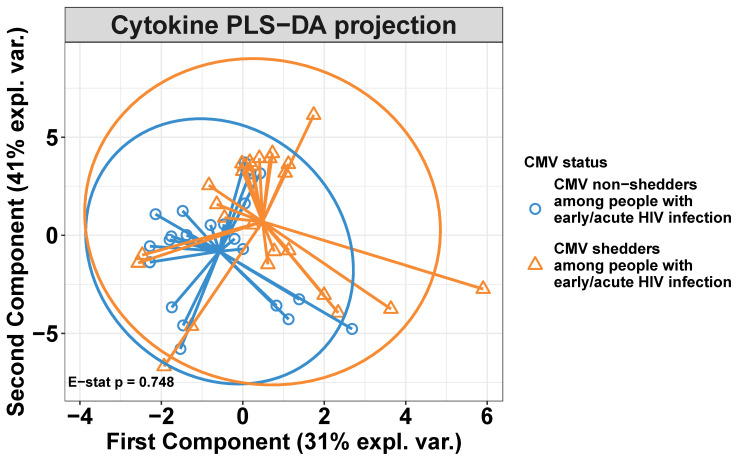
Two-dimensional PLS projections of blood cytokines in CMV shedders versus non-shedders. Shown are the PLS-DA projections in two LVs with ellipses representing Hotelling’s two-sample T2 with 95% confidence intervals in the blood plasma of people with early/acute HIV who shed CMV (orange triangles) and people who did not shed CMV (blue circles). PLS projections showed a large overlap between the cytokine profiles of CMV shedders versus non-shedders. The separation between the two groups, measured as the energy (E) statistic, was not statistically different (E-statistic *p* = 0.748).

Among PWH, 25 individuals shed seminal CMV, while 22 did not. We next investigated whether blood cytokines were influenced by seminal CMV shedding. Among all documented parameters, only CMV shedding was different (see Table 2). Similar to what was done for the previous analysis in predicting early/acute HIV-infection, the PLS-DA model used here was initially fitted with ten components to evaluate the performance and the number of components necessary for the optimal model. The best model was also obtained with two components or variables, although it was very poor. The error rate using two components was 45%, which is only slightly better than guessing at random (Figure S2). Samples projected into the subspace spanned by the two components are presented in Figure 2. PLS projections showed a large overlap between the cytokine profiles of CMV shedders versus non-shedders. The separation between the two groups was not statistically different (E-statistic *p* = 0.748) (Figure 2). Furthermore, none of the cytokines with the highest VIP (Table 5 and Table S4) was statistically different between CMV shedders and non-shedders according to Wilcoxon rank-sum testing (Table 6). Similar results were found in an independent individual cytokine analysis (Table S5).

In an attempt to compare each of the two PWH groups separately with the control group of people without HIV, we performed a new PLS-DA analysis based on a three-group model (people without HIV and CMV shedders or non-shedders among PWH). PLS projections showed distinct separation between the cytokine profiles of people without HIV and people with early/acute HIV whether they shed CMV or not. Moreover, there was no separation between CMV shedders and non-shedders in PWH (Figures S3 and S4). Furthermore, cytokines with VIP > 1 in the three-group analysis were similar to the ones obtained in the PLS-DA analysis of people with and without HIV (Table S6).

## 4. Discussion

CMV, a common β-herpesvirus, is one of the largest and most immunogenic viruses that infects humans. Despite its inflammatory potential, it rarely causes inflammatory conditions in immunocompetent individuals. In PWH however, CMV coinfection has been linked to non-AIDS comorbidities, such as cardiovascular disease, neurocognitive complications, cancer, fragility, and immunological aging (reviewed in [5,33,34]). The reason behind these adverse health outcomes is partly due to chronic activation as a result of CMV infection itself and the magnitude of the host’s immune response against CMV. Indeed, in PWH, CMV becomes the target of a large proportion of circulating T-cells, which skews the immune system toward a CMV-specific response, thus maintaining systemic immune activation/inflammation [35,36]. In PWH, despite suppressive ART, CMV has been associated with T-cell immunosenescence and immune exhaustion [11,18,23,24,37,38,39,40], slower decay of HIV DNA [41], and lower overall survival [20,42]. Furthermore, in PWH on ART, higher anti-CMV IgG antibody levels are associated with higher plasma levels of markers of gut damage translocation [40], and proinflammatory cytokines [16,17,18,19,20,21], including sCD14, a marker of monocyte activation. Although the associations between CMV replication and systemic inflammation in PWH during suppressive ART have been well documented, it not clear whether CMV replication is associated with systemic immune activation during the earliest phase of untreated HIV infection and therefore a potential target for early CMV intervention. Here, we investigated whether genital CMV shedding contributed to systemic immune activation, as evaluated by the concentration of 34 blood cytokines in PWH in the early/acute phase of HIV infection. We used PLS-DA, a supervised principal component-type analysis that is well adapted to datasets comprising a large number of variables (cytokines) [28,43].

First, we looked at the modulations of blood plasma cytokines associated with early/acute HIV infection independent of CMV. Our findings that plasma concentrations of IP-10, MIG, MCP-1, I-TAC, IL-16, and MIP-1β were modulated in early/acute HIV infection compared with people living without HIV are in agreement with previous reports [25,44,45]. Interestingly, IP-10, MIG, and I-TAC, which were upregulated here in early/acute phase, were the cytokines that were most downregulated in a study on the effect of early ART in PWH [43]. This particular finding for IP-10, MIG, and I-TAC was not surprising, as these three cytokines are all involved in Th1 trafficking in response to HIV infection via their receptor CXCR3.

In our cohort of people with early/acute HIV-infection, 53% of individuals shed CMV in semen, while 47% did not. Seminal CMV shedding is associated with higher seminal HIV levels in semen [26,46,47,48,49,50], contributing to higher risk of transmission [51,52]. However, little is known about the role of genital CMV shedding on systemic immune activation in PWH in early/acute phase. In particular, the effect of frequent asymptomatic CMV episodic bursts in semen on blood cytokines has not been addressed. Using PLS-DA, we found that PLS projections of blood cytokines in CMV shedders versus non-shedders strongly overlapped, suggesting that CMV replication in semen does not alter blood cytokines during early/acute HIV infection.

Although PLS-DA is considered particularly suitable for datasets with a large number of variables, such as cytokines, as used in our study, as an additional confirmatory analysis we compared all cytokines individually using a Wilcoxon rank-sum test in (i) people with early/acute HIV compared to people without HIV and (ii) in CMV shedders versus non-shedders among PWH. We found that the cytokines statistically different in the individual cytokine analysis were similar to the ones reported to be statistically different in our combined analysis of people with early/acute HIV compared to people without HIV (Table S5). Similarly, both PLS-DA and individual cytokine analysis showed that no cytokines were statically different between CMV shedders and non-shedders among PWH (Table S6). Altogether, these data validated our approach and our choice to apply PLS-DA to our dataset.

Positive correlations between the presence of CMV and soluble markers of inflammation and immune activation in blood have been previously reported in six studies [16,17,18,19,20,21]. However, all the studies correlated the markers of inflammation with CMV IgG antibody levels. Our study focused on CMV shedding and not IgG levels, as caution has been urged when considering CMV IgG antibodies as surrogate markers of active CMV replication [23,53]. For example, CMV replicates intermittently and may not have been “captured” in cross-sectional studies using CMV IgG. In fact, one of the studies reported on the association between CMV IgG and concentrations of sCD14 in blood although replicating CMV was not found [17]. CMV IgG levels or actual CMV replication aside, the differences in results between the six previous studies and our study may simply be related to the stage of HIV disease. While we investigated the effect of CMV shedding on systemic cytokines in early/acute HIV, all the other studies were performed in virologically suppressed or ART long-term PWH.

Our results suggest that CMV shedding in the male genital tract is not the main driver of systemic immune activation in the early phase of HIV infection. This contrasts with the later phase of HIV infection, in which CMV contributes to immune activation even when HIV replication is controlled on ART. Indeed, we and others have shown that asymptomatic shedding of CMV in the male genital tract is associated with increased systemic T-cell immune activation and proliferation and with higher levels of HIV DNA in peripheral CD4 T-cells [22,23,24].

We acknowledge certain limitations to our study. First, this is a cross-sectional study involving a single time point, which may not be fully reflective of CMV DNA and cytokine changes over time. Second, we did not have information on seminal cytokines and possible correlations with seminal shedding. Third, our study cohort was entirely composed of men. Our findings may not be relevant to women in whom frequent bursts of asymptomatic CMV reactivation have also been documented [54,55,56,57]. We acknowledge the evidence that the immune response to CMV may differ by sex [34]. For example, unlike what has been previously described for men, the presence of CMV DNA was not associated with increased HIV DNA in women [58]. Fourth, due to the limited sample size, the comparison between CMV shedders and non-shedders could be underpowered, thus limiting the statistical significance of the results. Finally, since absolute CD4 and CD8 T-cell counts were only available for PWH but not for controls, we provided CD4 and CD8 as percentages of total lymphocytes for all individuals for comparison.

Despite these limitations, to our knowledge, this is the first study on the effect of active CMV replication in semen on systemic cytokines in early/acute HIV infection. Our study may be one piece in the puzzle aiming at deciphering the exact role of CMV in contributing to persistent immune activation in PWH. Our results suggest that HIV rather than CMV replication in the male genital tract drives immune activation in the early phase of HIV infection. Thus, starting ART in early/acute HIV infection remains a priority to limit immune activation [59,60].

## Figures and Tables

**Table 1:** Demographic and clinical information: people with and without HIV.

	People without HIV	People with HIV	Overall	*p*-Value
**Race/ethnicity**	-	n = 47	n = 47	
**Hispanic/Latino**	-	10 (21.3%)	10 (21.3%)	-
**Other/Multiracial**	-	9 (19.1%)	9 (19.1%)	
**White (non-Hispanic)**	-	28 (59.6%)	28 (59.6%)	
**Age (years)**	n = 21	n = 48	n = 69	
	36.00 (25–69)	35.50 (19–54)	36.00 (19–69)	0.221
**Sex at birth**	n = 21	n = 48	n = 69	
**Male**	21 (100.0%)	48 (100.0%)	69 (100.0%)	-
**MSM**	n = 21	n = 48	n = 69	
**No**	4 (19.0%)	0 (0.0%)	4 (5.8%)	0.007 **
**Yes**	17 (81.0%)	48 (100.0%)	65 (94.2%)	
**On prep**	n = 21	n = 0	n = 21	
**No**	15 (71.4%)	0 (-)	15 (71.4%)	-
**Yes**	6 (28.6%)	0 (-)	6 (28.6%)	
**EDI to sample collection (weeks)**	N/A	n = 45	n = 45	
	-	12.14 (1.86, 153.00)	12.14 (1.86, 153.00)	-
**Nadir CD4 counts**	N/A	n = 48	n = 48	
	-	370.00 (120.00, 784.00)	370.00 (120.00, 784.00)	-
**Baseline CD4 counts**	N/A	n = 48	n = 48	
	-	460.00 (120.00, 959.00)	460.00 (120.00, 959.00)	-
**Baseline CD4/CD8 ratio**	N/A	n = 48	n = 48	
	-	0.60 (0.09, 1.61)	0.60 (0.09, 1.61)	-
**CD4 percentage at cytokine sample date**	n = 21	n = 48	n = 69	
	54.87 (26.62, 76.92)	36.03 (1.70, 61.71)	43.52 (1.70, 76.92)	<0.001 ***
**CD8 percentage at cytokine sample date**	n = 21	n = 48	n = 69	
	37.83 (19.57, 67.12)	53.39 (27.75, 78.70)	47.07 (19.57, 78.70)	0.002 **
**Peak viral load (log10)**	N/A	n = 48	n = 48	
	-	5.51 (3.78, 7.49)	5.51 (3.78, 7.49)	-
**Seminal CMV copies/mL (log10)**	N/A	n = 48	n = 48	
	-	2.35 (0.00, 8.38)	2.35 (0.00, 8.38)	-

Median (range) or count (percentage) shown for continuous or categorical variables respectively. *p*-values were calculated using a two-sample *t*-test for continuous variables, and a chi-square or Fisher’s exact test was used for categorical variables. EDI: Estimated Date of Infection. N/A: Not applicable. ** *p*-value < 0.01; *** *p*-value < 0.001.

**Table 2:** Demographic and clinical information: CMV shedders vs. non-shedders.

	Non-Shedders	Shedders	Overall	*p*-Value
**Race/ethnicity**	n = 22	n = 25	n = 47	
**Hispanic/Latino**	5 (22.7%)	5 (20.0%)	10 (21.3%)	0.716
**Other/Multiracial**	3 (13.6%)	6 (24.0%)	9 (19.1%)	
**White (non-Hispanic)**	14 (63.6%)	14 (56.0%)	28 (59.6%)	
**Age (years)**	n = 22	n = 26	n = 48	
	38.00 (23.00, 51.00)	32.50 (19.00, 54.00)	35.50 (19.00, 54.00)	0.443
**Sex at birth**	n = 22	n = 26	n = 48	
**Male**	22 (100.0%)	26 (100.0%)	48 (100.0%)	-
**MSM**	n = 22	n = 26	n = 48	
**Yes**	22 (100.0%)	26 (100.0%)	48 (100.0%)	-
**EDI to sample collection (weeks)**	n = 22	n = 23	n = 45	
	12.07 (3.00, 91.00)	12.14 (1.86, 153.00)	12.14 (1.86, 153.00)	0.895
**Nadir CD4 counts**	n = 22	n = 26	n = 48	
	379.00 (120.00, 731.00)	339.00 (143.00, 784.00)	370.00 (120.00, 784.00)	0.798
**Baseline CD4 counts**	n = 22	n = 26	n = 48	
	511.00 (120.00, 892.00)	412.00 (179.00, 959.00)	460.00 (120.00, 959.00)	0.51
**Baseline CD4/CD8 ratio**	n = 22	n = 26	n = 48	
	0.79 (0.14, 1.52)	0.43 (0.09, 1.61)	0.60 (0.09, 1.61)	0.081
**CD4 percentage at cytokine sample date**	n = 22	n = 26	n = 48	
	41.53 (22.67, 61.71)	31.05 (1.70, 55.14)	36.03 (1.70, 61.71)	0.009 **
**CD8 percentage at cytokine sample date**	n = 22	n = 26	n = 48	
	46.45 (29.16, 63.42)	55.05 (27.75, 78.70)	53.39 (27.75, 78.70)	0.056
**Peak HIV viral load (log10)**	n = 22	n = 26	n = 48	
	5.41 (3.78, 7.49)	5.85 (4.42, 7.44)	5.51 (3.78, 7.49)	0.362
**Seminal CMV copies/mL (log10)**	n = 22	n = 26	n = 48	
	N/A	4.73 (2.00, 8.38)	2.35 (0.00, 8.38)	<0.001 ***

Median (range) or count (percentage) shown for continuous or categorical variables, respectively. *p*-values were calculated using a two-sample *t*-test for continuous variables, and a chi-square or Fisher’s exact test was used for categorical variables. EDI: Estimated Date of Infection. N/A: Not applicable. ** *p*-value < 0.01; *** *p*-value < 0.001.

**Table 3:** VIP scores for each cytokine: people with and without HIV.

Cytokine	VIP
IP-10	1.65
MCP-1	1.39
MIP-1β	1.28
GM-CSF	1.25
MIG	1.24
IL-18	1.16
ITAC	1.16
IL-17	1.15
RANTES	1.13
IL-16	1.06
EOTAXIN	1.04
TNF-α	1.04
IL-7	0.96
IL-4	0.95
CAL	0.92
IL-1β	0.86
TGF-β	0.8
GRO-α	0.79
IL-21	0.78
IL-15	0.75
IL-12	0.71
CMVIL-10	0.7
MIP-1α	0.7
IL-6	0.69
IFN-γ	0.67
IL-22	0.65
M-CSF	0.58

**Table 4:** Results from Wilcoxon rank-sum test: people with and without HIV.

Cytokine	Raw *p*-Value	Adjusted *p*-Value
RANTES	0.872	0.872
TNF-α	0.469	0.512
Eotaxin	0.234	0.28
GM-CSF	0.059	0.078
IL-17	0.047 *	0.075
IL-18	0.05	0.075
MIP-1β	0.018 *	0.035 *
IL-16	0.004 **	0.01 **
ITAC	0.004 **	0.01 **
MCP-1	0.002 **	0.008 **
MIG	<0.001 ***	<0.001 ***
IP-10	<0.001 ***	<0.001 ***

* *p*-value < 0.05; ** *p*-value < 0.01; *** *p*-value < 0.001.

**Table 5:** VIP scores for each cytokine: CMV shedders versus non-shedders.

Cytokine	VIP
RANTES	1.48
IL-18	1.42
IL-17	1.33
IL-16	1.3
IL-6	1.29
IP-10	1.28
MIP-1β	1.19
ITAC	1.16
MCP-1	1.13
GRO-α	1.04
IL-7	1.04
IL-15	1
IL-4	0.9
MIG	0.9
EOTAXIN	0.87
IL-22	0.85
MIP-1α	0.82
CMVIL-10	0.81
GM-CSF	0.81
IL-1β	0.76
TGF-β	0.71
CAL	0.7
IFN-γ	0.7
IL-12	0.7
M-CSF	0.67
IL-21	0.63
TNF-α	0.6

**Table 6:** Results from Wilcoxon rank-sum test: CMV shedders versus non-shedders.

Cytokine	Raw *p*-Value	Adjusted *p*-Value
GRO-α	0.872	0.959
IL-16	0.786	0.959
IL-18	0.858	0.959
IL-6	0.932	0.959
IL-7	0.66	0.959
MCP-1	0.8	0.959
RANTES	0.959	0.959
IL-17	0.263	0.722
IP-10	0.146	0.535
ITAC	0.103	0.535
MIP-1β	0.104	0.535

## Data Availability

Data available upon request.

## Supplementary Materials

The following supporting information can be downloaded at: https://www.mdpi.com/article/10.3390/v14081833/s1, Figure S1: Classification error for people with early/acute HIV or people without HIV in PLSDA model estimated using k-fold CV; Figure S2: Classification error for CMV shedders versus CMV non-shedders in PLSDA model estimated using k-fold CV; Figure S3: Two-dimensional PLS projections of blood cytokines in people without HIV and in CMV shedders versus non-shedders among people with early/acute HIV; Figure S4: The statistical significance of 12 cytokines with VIP > 1 identified in the PLS-DA model (comparing people with early/acute HIV versus people without HIV) were tested by Wilcoxon Rank Sum test. Table S1: Lower limits of detection for each Cytokine included in the assay and percentage of participants with undetectable values for each cytokine split the respective analysis; Table S2: Median (range) of log-transformed and normalized concentrations of cytokine with VIP > 1 in people with early HIV infection and people without HIV; Table S3: Median (range) for each log-normalized cytokine in people with early/acute HIV and people without HIV. Cytokines were individually tested using Wilcoxon rank-sum test to compare groups; Table S4: Median (range) of log-transformed and normalized concentrations of cytokine with VIP > 1 in CMV shedders versus non-shedders; Table S5: Median (range) for each log-normalized cytokine in CMV shedders vs non-shedders among people with early/acute HIV. Cytokines were individually tested using Wilcoxon rank-sum test to compare groups; Table S6: VIP scores for each cytokine in a PLS-DA 3-group model: people without HIV and CMV shedders versus non shedders among people with early/acute HIV.

## Abbreviations

HIV	Human Immunodeficiency Virus
CMV	Cytomegalovirus
PLS-DA	Partial least squares-discriminant analysis
MSM	Men who have sex with men
