# Supplementary material for: HIV but Not CMV Replication Alters the Blood Cytokine Network during Early HIV Infection in Men
Source: Viruses. 2022 Aug 21;14(8):1833. doi: 10.3390/v14081833 (PMC9416553; doi:10.3390/v14081833)
Supplement: Supplementary file 1 [file viruses-14-01833-s001.zip › viruses-1854599-supplemental.pdf]

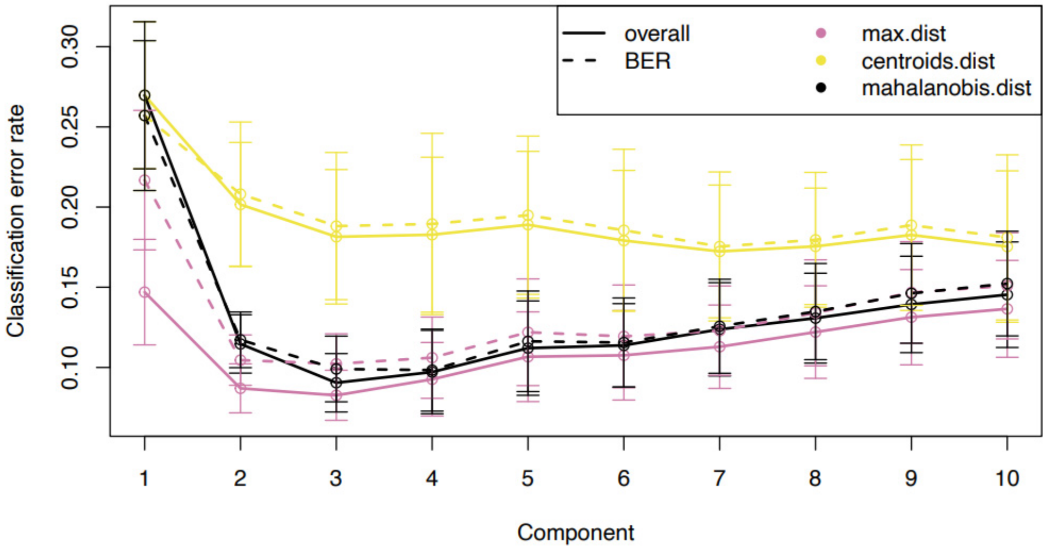

Figure S1

Legend to Supplemental Figure S1: Classification error for people with early/acute HIV or people without HIV in PLSDA model estimated using k-fold CV.

The classification performance of the PLS-DA model was assessed with the perf function using 5-fold cross-validation repeated 100 times. The number of repeats is necessary to ensure a good estimate of the classification error rate (as the CV-folds are determined in a random manner). A PLS-DA model was initially fitted with ten components to evaluate the performance and the number of components necessary for the final model. From the performance results we can decide on the choice of the number of components of the final PLS model.

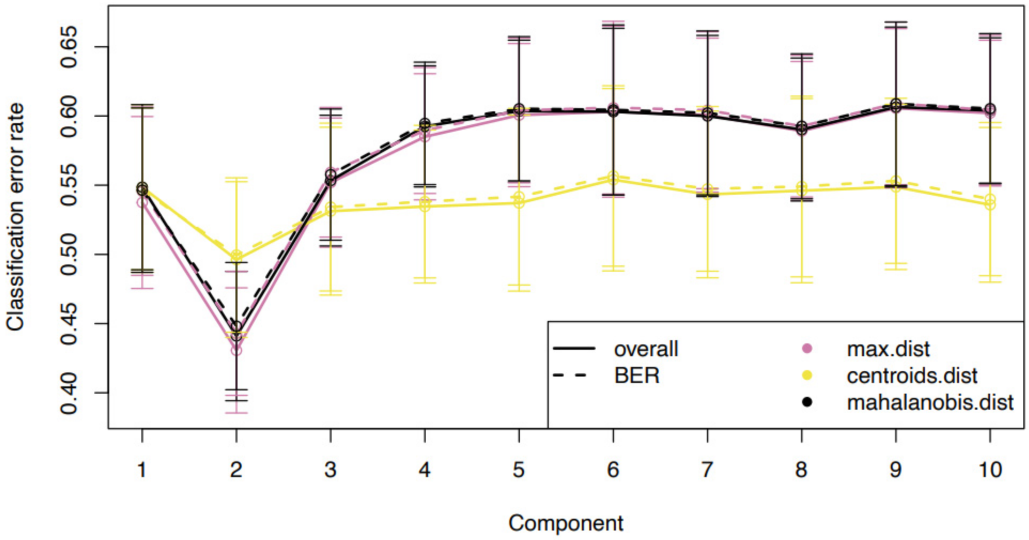

Figure S2

Legend to Supplemental Figure S2: Classification error for CMV shedders versus CMV non-shedders in PLSDA model estimated using k-fold CV.

Similar to the classification error for the PLSDA model between people with early/acute HIV and people without HIV, the classification performance of the PLS-DA model for CMV shedders vs CMV non-shedders was assessed with the perf function using 5-fold cross-validation repeated 100 times. The number of repeats is necessary to ensure a good estimate of the classification error rate. A PLS-DA model was initially fitted with ten components to evaluate the performance and the number of components necessary for the final model.

From the performance plot, we observe that the overall error rate and the Balanced Error Rate were poor for CMV shedders vs non-shedders. The minimum error rate was obtained using 2 components and was only 45%, which is only slightly better than guessing at random.

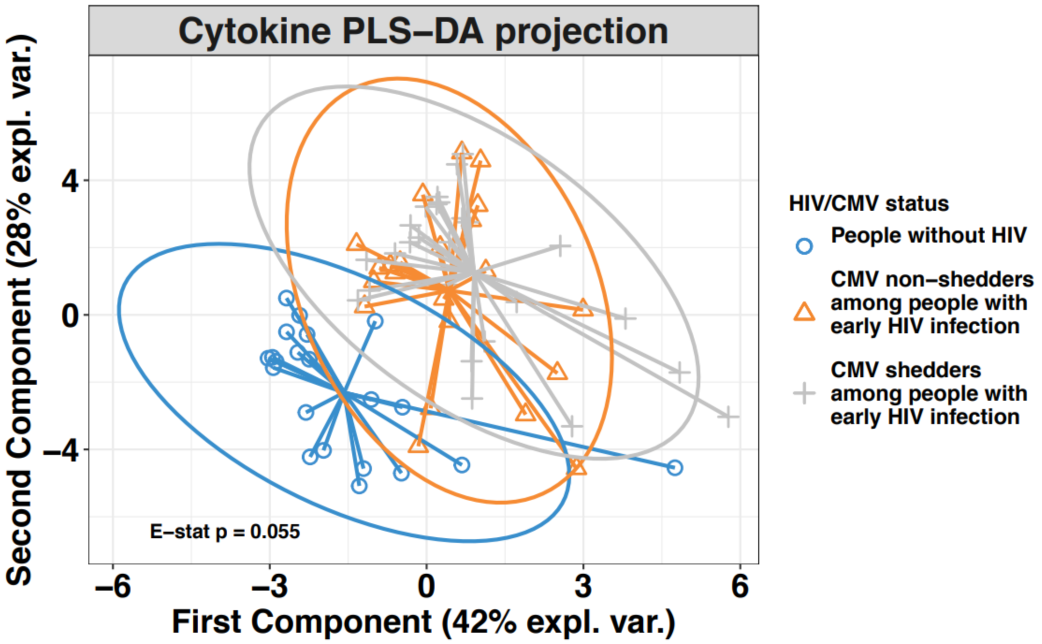

Figure S3

Legend to Supplemental Figure S3: Two-dimensional PLS projections of blood cytokines in people without HIV and in CMV shedders versus non-shedders among people with early/acute HIV.

Shown are PLS-DA projections in two LVs with ellipses representing Hotelling's 2-samples T2 with 95% confidence intervals in blood plasma for people with HIV in the early/acute HIV phase shedding CMV (grey crosses) or not (orange triangles) or in people without HIV (blue circles). The E-statistic was used to test the statistical differences in the separation between the cytokine profiles of the two groups. The multivariate distance between the 3 groups was not significant (*p* = 0.055).

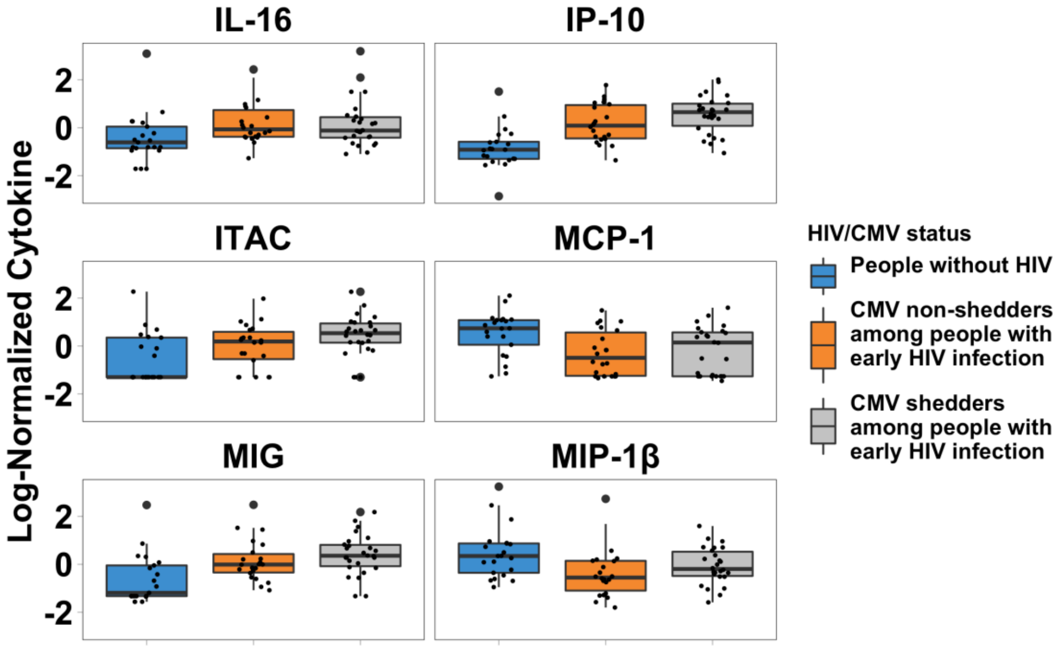

Figure S4

Legend to Supplemental Figure S4: The statistical significance of 12 cytokines with VIP>1 identified in the PLS-DA model (comparing people with early/acute HIV versus people without HIV) were tested by Wilcoxon Rank Sum test. Six out of 12 remained significant. Shown is the difference of the log_{10}-transformed concentrations plotted as boxplots of the 6 chemokine/cytokines in people with HIV in the early/acute phase shedding CMV or not and in people without HIV. For each cytokine and each boxplot, each point represents a participant's cytokine concentration, the box represents the interquartile range (IQR), the middle line represents the median, while the points beyond the whiskers are outliers. Note that these cytokines were not significant in the 3-group model (when comparing PWH with and without CMV shedding).

Supplementary Table S1: Lower limits of detection for each Cytokine included in the assay and percentage of participants with undetectable values for each cytokine split the respective analysis.

cytokine	Lower limit of detection (pg/mL)	Undetectable amongst people with and without HIV (%)	Undetectable amongst CMV shedders/non-shedders (%)
**IL-10**	9.731	81.16	79.17
**MIP-3α**	6.138	81.16	81.25
**GRO-α**	2.2	44.93	43.75
**Cal**	3.028	59.42	64.58
**IL-33**	41.589	91.3	89.58
**IL-21**	44.622	0	0
**IL-12**	1.207	24.64	22.92
**ITAC**	3.92	28.99	16.67
**IL-18**	0.268	1.45	2.08
**IL-4**	0.433	62.32	62.5
**IFN-γ**	8.219	52.17	54.17
**IL-8**	1.553	79.71	79.17
**IL-16**	27.445	0	0
**IL-2**	2.7	85.51	81.25
**GM-CSF**	13.129	40.58	58.33
**M-CSF**	1.012	50.72	45.83
**IP-10**	6.978	0	0
**TNF-α**	0.35	15.94	6.25
**IL-1α**	0.466	72.46	70.83
**IL-6**	0.684	56.52	54.17
**IL-15**	3.083	42.03	45.83
**RANTES**	1.88	0	0
**TGF-b**	1.064	55.07	52.08
**MIP-1α**	0.68	43.48	41.67
**MIG**	3338.2	11.59	4.17
**MCP-1**	0.65	18.84	25

**IL-1β**	7.432	27.54	31.25
**Eotaxin**	7.223	1.45	0
**IL-13**	69.678	84.06	81.25
**IL-17**	8.853	68.12	58.33
**IL-22**	87.53	55.07	54.17
**IL-7**	0.416	33.33	27.08
**MIP-1β**	0.354	0	0
**cmvIl-10**	1.268	18.84	14.58

Supplementary Table S2: Median (range) of log-transformed and normalized concentrations of cytokine with VIP > 1 in people with early HIV infection and people without HIV.

	People without HIV (n = 21)	People with HIV (n = 48)	Overall (n = 69)
**Eotaxin**	-0.02 (-4.34, 2.33)	-0.21 (-1.22, 4.44)	-0.13 (-4.34, 4.44)
**GM-CSF**	-0.08 (-1.81, 3.10)	-0.76 (-0.76, 3.24)	-0.22 (-1.81, 3.24)
**IL-16**	-0.61 (-1.71, 3.09)	-0.12 (-1.27, 3.19)	-0.21 (-1.71, 3.19)
**IL-17**	-0.55 (-0.55, 3.02)	-0.55 (-0.82, 3.41)	-0.55 (-0.82, 3.41)
**IL-18**	-0.24 (-2.11, 2.19)	0.10 (-3.84, 2.83)	0.02 (-3.84, 2.83)
**IP-10**	-0.92 (-2.86, 1.51)	0.44 (-1.36, 2.01)	-0.06 (-2.86, 2.01)
**ITAC**	-1.30 (-1.30, 2.27)	0.33 (-1.30, 2.26)	0.16 (-1.30, 2.27)
**MCP-1**	0.74 (-1.27, 2.10)	-0.25 (-1.46, 1.60)	0.16 (-1.46, 2.10)
**MIG**	-1.19 (-1.56, 2.47)	0.22 (-1.33, 2.48)	-0.01 (-1.56, 2.48)
**MIP-1β**	0.34 (-0.95, 3.24)	-0.33 (-1.80, 2.73)	-0.16 (-1.80, 3.24)
**RANTES**	0.15 (-2.53, 1.59)	0.05 (-2.04, 1.82)	0.05 (-2.53, 1.82)
**TNF-α**	0.02 (-1.18, 2.16)	-0.20 (-1.25, 2.01)	-0.12 (-1.25, 2.16)

Supplementary Table S3: Median (range) for each log-normalized cytokine in people with early/acute HIV and people without HIV. Cytokines were individually tested using Wilcoxon rank-sum test to compare groups.

	**HIV negative (n = 21)**	**HIV positive (acute phase) (n = 48)**	**Overall (n = 69)**	**P-value (raw)**	**Adjusted P-value**
Cal	-0.42 (-0.71, 2.09)	-0.71 (-1.05, 2.15)	-0.71 (-1.05, 2.15)	0.221	0.484
cmvIl-10	-0.54 (-1.24, 2.05)	-0.01 (-1.33, 2.52)	-0.11 (-1.33, 2.52)	0.148	0.4
Eotaxin	-0.02 (-4.34, 2.33)	-0.21 (-1.22, 4.44)	-0.13 (-4.34, 4.44)	0.233	0.484
GM-CSF	-0.08 (-1.81, 3.10)	-0.76 (-0.76, 3.24)	-0.22 (-1.81, 3.24)	0.06	0.18
GRO-α	-0.78 (-0.99, 1.64)	0.05 (-0.99, 1.78)	0.01 (-0.99, 1.78)	0.738	0.869
IFN-γ	-0.72 (-1.24, 2.70)	-0.72 (-0.93, 2.67)	-0.72 (-1.24, 2.70)	0.773	0.869
IL-12	-0.10 (-1.30, 1.85)	0.07 (-1.24, 1.84)	0.01 (-1.30, 1.85)	0.427	0.761
IL-15	-0.34 (-1.01, 2.84)	-0.40 (-1.18, 3.57)	-0.34 (-1.18, 3.57)	0.65	0.869
IL-16	-0.61 (-1.71, 3.09)	-0.12 (-1.27, 3.19)	-0.21 (-1.71, 3.19)	0.004**	0.027*
IL-17	-0.55 (-0.55, 3.02)	-0.55 (-0.82, 3.41)	-0.55 (-0.82, 3.41)	0.048*	0.172
IL-18	-0.24 (-2.11, 2.19)	0.10 (-3.84, 2.83)	0.02 (-3.84, 2.83)	0.051	0.172
IL-1β	0.24 (-1.25, 1.96)	-0.01 (-1.25, 2.18)	-0.00 (-1.25, 2.18)	0.777	0.869
IL-21	-0.10 (-1.82, 1.97)	-0.18 (-1.44, 2.26)	-0.13 (-1.82, 2.26)	0.527	0.79
IL-22	-0.81 (-0.81, 2.65)	-0.81 (-0.81, 2.88)	-0.81 (-0.81, 2.88)	0.479	0.761
IL-4	-0.57 (-0.57, 2.99)	-0.57 (-0.57, 2.81)	-0.57 (-0.57, 2.99)	0.805	0.869
IL-6	-0.61 (-0.61, 3.12)	-0.61 (-0.73, 3.29)	-0.61 (-0.73, 3.29)	0.703	0.869

IL-7	-0.72 (-0.90, 2.79)	-0.09 (-0.90, 2.93)	-0.14 (-0.90, 2.93)	0.165	0.405
IP-10	-0.92 (-2.86, 1.51)	0.44 (-1.36, 2.01)	-0.06 (-2.86, 2.01)	< .001***	0.013*
ITAC	-1.30 (-1.30, 2.27)	0.33 (-1.30, 2.26)	0.16 (-1.30, 2.27)	0.005**	0.027*
M-CSF	-0.80 (-0.80, 2.51)	-0.49 (-0.80, 2.73)	-0.80 (-0.80, 2.73)	0.374	0.721
MCP-1	0.74 (-1.27, 2.10)	-0.25 (-1.46, 1.60)	0.16 (-1.46, 2.10)	0.002**	0.018*
MIG	-1.19 (-1.56, 2.47)	0.22 (-1.33, 2.48)	-0.01 (-1.56, 2.48)	< .001***	0.013*
MIP-1α	0.35 (-0.97, 2.03)	-0.17 (-0.97, 2.11)	-0.16 (-0.97, 2.11)	0.984	0.984
MIP-1β	0.34 (-0.95, 3.24)	-0.33 (-1.80, 2.73)	-0.16 (-1.80, 3.24)	0.019*	0.085
RANTES	0.15 (-2.53, 1.59)	0.05 (-2.04, 1.82)	0.05 (-2.53, 1.82)	0.872	0.906
TGF-b	-0.77 (-0.77, 2.39)	-0.77 (-0.77, 2.39)	-0.77 (-0.77, 2.39)	0.568	0.807
TNF-α	0.02 (-1.18, 2.16)	-0.20 (-1.25, 2.01)	-0.12 (-1.25, 2.16)	0.468	0.761

Supplementary Table S4: Median (range) of log-transformed and normalized concentrations of cytokine with VIP > 1 in CMV shedders versus non-shedders.

	**Non-shedders (n = 22)**	**Shedders (n = 26)**	**Overall (n = 48)**
**GRO-α**	0.16 (-0.99, 1.58)	0.02 (-0.99, 1.78)	0.05 (-0.99, 1.78)
**IL-16**	-0.07 (-1.27, 2.43)	-0.12 (-1.10, 3.19)	-0.12 (-1.27, 3.19)
**IL-17**	-0.55 (-0.55, 1.66)	-0.55 (-0.82, 3.41)	-0.55 (-0.82, 3.41)
**IL-18**	0.15 (-3.84, 1.17)	0.08 (-0.85, 2.83)	0.10 (-3.84, 2.83)
**IL-6**	-0.61 (-0.61, 2.05)	-0.61 (-0.73, 3.29)	-0.61 (-0.73, 3.29)
**IL-7**	-0.03 (-0.90, 1.60)	-0.22 (-0.90, 2.93)	-0.09 (-0.90, 2.93)
**IP-10**	0.09 (-1.36, 1.78)	0.65 (-1.06, 2.01)	0.44 (-1.36, 2.01)
**ITAC**	0.19 (-1.30, 1.98)	0.53 (-1.30, 2.26)	0.33 (-1.30, 2.26)
**MCP-1**	-0.49 (-1.34, 1.49)	0.14 (-1.46, 1.60)	-0.25 (-1.46, 1.60)
**MIP-1β**	-0.55 (-1.80, 2.73)	-0.20 (-1.59, 1.60)	-0.33 (-1.80, 2.73)
**RANTES**	0.06 (-2.02, 1.51)	0.00 (-2.04, 1.82)	0.05 (-2.04, 1.82)

Supplementary Table S5: Median (range) for each log-normalized cytokine in CMV shedders vs non-shedders among people with early/acute HIV. Cytokines were individually tested using Wilcoxon rank-sum test to compare groups.

	**HIV positive (acute phase) / CMV non-shedders (n = 22)**	**HIV positive (acute phase) / CMV shedders (n = 26)**	**Overall (n = 48)**	**P-value (raw)**	**Adjusted P-value**
Cal	-0.71 (-1.05, 2.03)	-0.71 (-0.71, 2.15)	-0.71 (-1.05, 2.15)	0.971	0.971
cmvIl-10	0.12 (-1.33, 2.52)	-0.09 (-1.24, 2.36)	-0.01 (-1.33, 2.52)	0.583	0.971
Eotaxin	-0.31 (-0.91, 1.23)	-0.07 (-1.22, 4.44)	-0.21 (-1.22, 4.44)	0.492	0.971
GM-CSF	-0.76 (-0.76, 1.68)	-0.76 (-0.76, 3.24)	-0.76 (-0.76, 3.24)	0.936	0.971
GRO-α	0.16 (-0.99, 1.58)	0.02 (-0.99, 1.78)	0.05 (-0.99, 1.78)	0.871	0.971
IFN-γ	-0.72 (-0.72, 1.85)	-0.72 (-0.93, 2.67)	-0.72 (-0.93, 2.67)	0.857	0.971
IL-12	0.17 (-1.24, 1.84)	-0.10 (-1.24, 1.82)	0.07 (-1.24, 1.84)	0.411	0.971
IL-15	-0.22 (-1.18, 1.71)	-0.82 (-0.82, 3.57)	-0.40 (-1.18, 3.57)	0.905	0.971
IL-16	-0.07 (-1.27, 2.43)	-0.12 (-1.10, 3.19)	-0.12 (-1.27, 3.19)	0.788	0.971
IL-17	-0.55 (-0.55, 1.66)	-0.55 (-0.82, 3.41)	-0.55 (-0.82, 3.41)	0.262	0.971
IL-18	0.15 (-3.84, 1.17)	0.08 (-0.85, 2.83)	0.10 (-3.84, 2.83)	0.86	0.971
IL-1β	0.11 (-1.25, 2.18)	-0.03 (-1.25, 1.82)	-0.01 (-1.25, 2.18)	0.651	0.971
IL-21	-0.07 (-1.44, 2.26)	-0.31 (-1.35, 2.06)	-0.18 (-1.44, 2.26)	0.583	0.971
IL-22	-0.39 (-0.81, 1.38)	-0.81 (-0.81, 2.88)	-0.81 (-0.81, 2.88)	0.883	0.971
IL-4	-0.57 (-0.57, 2.65)	-0.57 (-0.57, 2.81)	-0.57 (-0.57, 2.81)	0.896	0.971
IL-6	-0.61 (-0.61, 2.05)	-0.61 (-0.73, 3.29)	-0.61 (-0.73, 3.29)	0.937	0.971

IL-7	-0.03 (-0.90, 1.60)	-0.22 (-0.90, 2.93)	-0.09 (-0.90, 2.93)	0.661	0.971
IP-10	0.09 (-1.36, 1.78)	0.65 (-1.06, 2.01)	0.44 (-1.36, 2.01)	0.146	0.971
ITAC	0.19 (-1.30, 1.98)	0.53 (-1.30, 2.26)	0.33 (-1.30, 2.26)	0.103	0.971
M-CSF	-0.41 (-0.80, 1.73)	-0.69 (-0.80, 2.73)	-0.49 (-0.80, 2.73)	0.802	0.971
MCP-1	-0.49 (-1.34, 1.49)	0.14 (-1.46, 1.60)	-0.25 (-1.46, 1.60)	0.802	0.971
MIG	-0.01 (-1.08, 2.48)	0.36 (-1.33, 2.18)	0.22 (-1.33, 2.48)	0.121	0.971
MIP-1α	-0.07 (-0.97, 1.87)	-0.68 (-0.97, 2.11)	-0.17 (-0.97, 2.11)	0.621	0.971
MIP-1β	-0.55 (-1.80, 2.73)	-0.20 (-1.59, 1.60)	-0.33 (-1.80, 2.73)	0.104	0.971
RANTES	0.06 (-2.02, 1.51)	0.00 (-2.04, 1.82)	0.05 (-2.04, 1.82)	0.959	0.971
TGF-b	-0.55 (-0.77, 1.81)	-0.77 (-0.77, 2.39)	-0.77 (-0.77, 2.39)	0.964	0.971
TNF-α	-0.10 (-1.18, 1.63)	-0.26 (-1.25, 2.01)	-0.20 (-1.25, 2.01)	0.521	0.971

Supplementary Table S6: VIP scores for each cytokine in a PLS-DA 3-group model: people without HIV and CMV shedders versus non shedders among people with early/acute HIV.

	VIP
**MCP-1**	1.35
**IL-18**	1.34
**IP-10**	1.33
**IL-16**	1.27
**IL-17**	1.27
**RANTES**	1.26
**EOTAXIN**	1.2
**GM-CSF**	1.2
**IL-6**	1.2
**MIP-1β**	1.14
**IL-4**	1.06

**ITAC**	1
**IL-7**	0.96
**CAL**	0.91
**TGF-B**	0.91
**GRO-α**	0.9
**MIG**	0.89
**TNF-α**	0.81
**IL-1β**	0.77
**IFN-γ**	0.74
**MIP-1α**	0.73
**IL-22**	0.72
**CMVIL-10**	0.7
**IL-12**	0.7
**IL-21**	0.67
**IL-15**	0.62
**M-CSF**	0.5
